# A Ternary Seismic Metamaterial for Low Frequency Vibration Attenuation

**DOI:** 10.3390/ma15031246

**Published:** 2022-02-08

**Authors:** Chen Chen, Jincheng Lei, Zishun Liu

**Affiliations:** International Center for Applied Mechanics, State Key Laboratory for Strength and Vibration of Mechanical Structures, School of Aerospace Engineering, Xi’an Jiaotong University, Xi’an 710049, China; cc19971120@stu.xjtu.edu.cn (C.C.); jinchenglei@mail.xjtu.edu.cn (J.L.)

**Keywords:** seismic metamaterial, band gap, low-frequency vibration attenuation, equivalent mass–spring model

## Abstract

Structural vibration induced by low frequency elastic waves presents a great threat to infrastructure such as buildings, bridges, and nuclear structures. In order to reduce the damage of low frequency structural vibration, researchers proposed the structure of seismic metamaterial, which can be used to block the propagation of low frequency elastic wave by adjusting the frequency range of elastic wave propagation. In this study, based on the concept of phononic crystal, a ternary seismic metamaterial is proposed to attenuate low frequency vibration by generating band gaps. The proposed metamaterial structure is periodically arranged by cube units, which consist of rubber coating, steel scatter, and soft matrix (like soil). The finite element analysis shows that the proposed metamaterial structure has a low frequency band gap with 8.5 Hz bandwidth in the range of 0–20 Hz, which demonstrates that the metamaterial can block the elastic waves propagation in a fairly wide frequency range within 0–20 Hz. The frequency response analysis demonstrates that the proposed metamaterial can effectively attenuate the low frequency vibration. A simplified equivalent mass–spring model is further proposed to analyze the band gap range which agrees well with the finite element results. This model provides a more convenient method to calculate the band gap range. Combining the proposed equivalent mass–spring model with finite element analysis, the effect of material parameters and geometric parameters on the band gap characteristic is investigated. This study can provide new insights for low frequency vibration attenuation.

## 1. Introduction

Transportation, construction, or earthquakes can induce the propagation of low frequency elastic waves near structures [1]. The structural vibration and machine noise generated by low frequency elastic wave can not only destroy civil infrastructure, but also reduce the comfort level of residents [2,3]. Among all the negative impacts caused by low frequency elastic waves, the damage to human society and environment induced by seismic waves is the most destructive. Traditional approaches for seismic resistance, mostly in the form of structural anti-seismic and seismic isolation, have certain limitations [4,5,6,7]. They often induce large movement of the structures which may not be acceptable. In other words, they cannot take effect before the seismic wave reaches the structures to block the seismic wave from the propagation path. Seismic metamaterials, with their inherent ability to manipulate low frequency elastic wave propagation, provide a key route for overcoming this challenge [8,9,10,11,12,13]. Based on the concept of phononic crystal, seismic metamaterials can block the propagation of elastic waves in a certain frequency range, which is called band gap [14,15,16,17,18,19,20]. Therefore, seismic metamaterial shows broad development prospects for the low-frequency vibration attenuation induced by elastic wave [21,22,23,24]. Local resonance phononic crystal proposed by Liu et al. provided a new idea to design seismic metamaterial for low frequency vibration attenuation [25]. Chen et al. analyzed the band gap characteristics of seismic metamaterial under different lattice types [26]. Ungureanu et al. proposed a negative Poisson’s ratio structure, which also has a great attenuation effect on low frequency vibration between 30 Hz and 50 Hz [27]. In addition, seismic metamaterial shows broad application prospects in other fields. Huang et al. found that the elastic wave metamaterial has more powerful energy barrier at low crack speeds, which demonstrates that the seismic metamaterial has great application potential in improving structural strength like resisting cracking [28]. Sadat et al. use the machine learning to predict the band gap characteristic of phononic crystal, which demonstrates the utility of machine learning for seismic metamaterial property discovery [29].

The experiment of seismic metamaterials can be divided into two types according to the experimental scale. One is the full-scale outdoor experiment. The first large-scale outdoor experiment of seismic metamaterials is the low-frequency vibration attenuation experiment of the soil drilling array [30]. It successfully proves the feasibility of seismic metamaterials at common scales. Liu et al. verified the vibration attenuation effect of the single-row concrete pile by outdoor vibration test [31]. Colombi et al. took forest as the seismic metamaterial and carried out seismic geophysics experiments [32]. Yan et al. designed a 2D metamaterial foundation and verified its isolation effect on elastic waves through the outdoor field tests [33]. Due to the strict requirement, the outdoor experiment is not easy to realize. Therefore, the indoor equivalent experiment by reducing the size of metamaterial has been carried out continuously. Witarto et al. carried out the vibration attenuation test of 1D metamaterial foundation and Chen et al. carried out the laboratory scale experiment for evaluating the vibration attenuation of layered soil metamaterial [34,35]. The development of experimental methods contributes to the verification of the vibration attenuation performance of seismic metamaterials.

The development of finite element analysis lays the foundation for the numerical simulation of seismic metamaterials. Guo summarized the finite element models and analysis methods of composite materials, which provides an important reference for the constitutive model optimization of seismic metamaterials [36]. Khalid provides a simple and effective estimation method for the free-edge interlaminar stress distribution under composite load, which is an inspiration for the calculation of stress distribution of seismic metamaterials under external load [37]; Lee et al. proposed a hybrid method of finite element analysis and empirical modeling to obtain the dynamic stiffness of rubber bushing in a wide excitation frequency and amplitude range, which provides an idea for the calculation of the equivalent stiffness of the components of seismic metamaterials [38]. The finite element analysis methods are an important tool in obtaining the band gap and evaluating the vibration attenuation performance of seismic metamaterials.

Based on the development of experiment methods and finite element analysis, many seismic metamaterials with lower band gap frequency were designed to attenuate the low-frequency vibration. Achaoui et al. designed a resonant array to obtain the band gap frequency between 16 Hz and 21 Hz. They further created the ultra-low frequency band gap by clamping the steel inclusions to a bed rock which lies underneath a soil layer [39,40]. Miniaci et al. studied the band gap characteristic below 10 Hz of different hollow-shaped seismic metamaterials [41]. Krödel et al. proposed a seismic metamaterial with cylindrical tubes array to obtain the band gap between 4 Hz and 7 Hz [42]. In order to attenuate vibration in a larger low-frequency range, Zeng et al. designed a cylinder seismic metamaterial with rubber rings and realized a low frequency bandwidth of 7 Hz [43]. Xu et al. realized the band gap range of 8–16 Hz by designing a H-shaped seismic metamaterial [44]. In addition, some natural objects show the potential to be precisely designed as seismic metamaterials. For example, Colombi et al. designed the forests as wedge arrays to shield the low-frequency components of elastic wave by means of wave reflection and modal transformation [32,45]. Although most of the above seismic metamaterials can block the low-frequency elastic waves below 20 Hz, the bandwidth of the band gap is not large enough. Proposing an effective method to increase the seismic metamaterial bandwidth in low-frequency range is still an important research topic.

In this study, we proposed a ternary seismic metamaterial to attenuate low frequency vibration below 20 Hz. In Section 2, we present the structure model of the proposed metamaterial and the dispersion analysis for band gap calculation in finite element method (FEM). In Section 3, the band gap calculation and the frequency response analysis of the metamaterial is carried out using FEM. In Section 4, we propose an equivalent mass–spring model to theoretically analyze the band gap. In Section 5, we further investigate the effect of geometric parameters and material parameters of the metamaterial on the band gap distribution for band gap optimization. Conclusions are presented in Section 6.

## 2. Metamaterial Model and Dispersion Analysis

In this section, we propose the structure model of the seismic metamaterial and present the dispersion analysis process for calculating the band gap characteristics.

**Figure 1:**
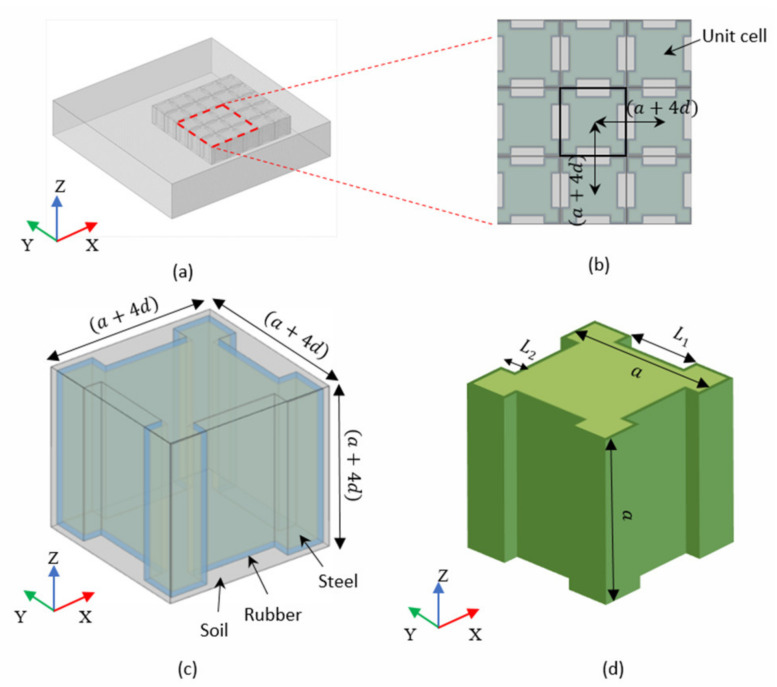

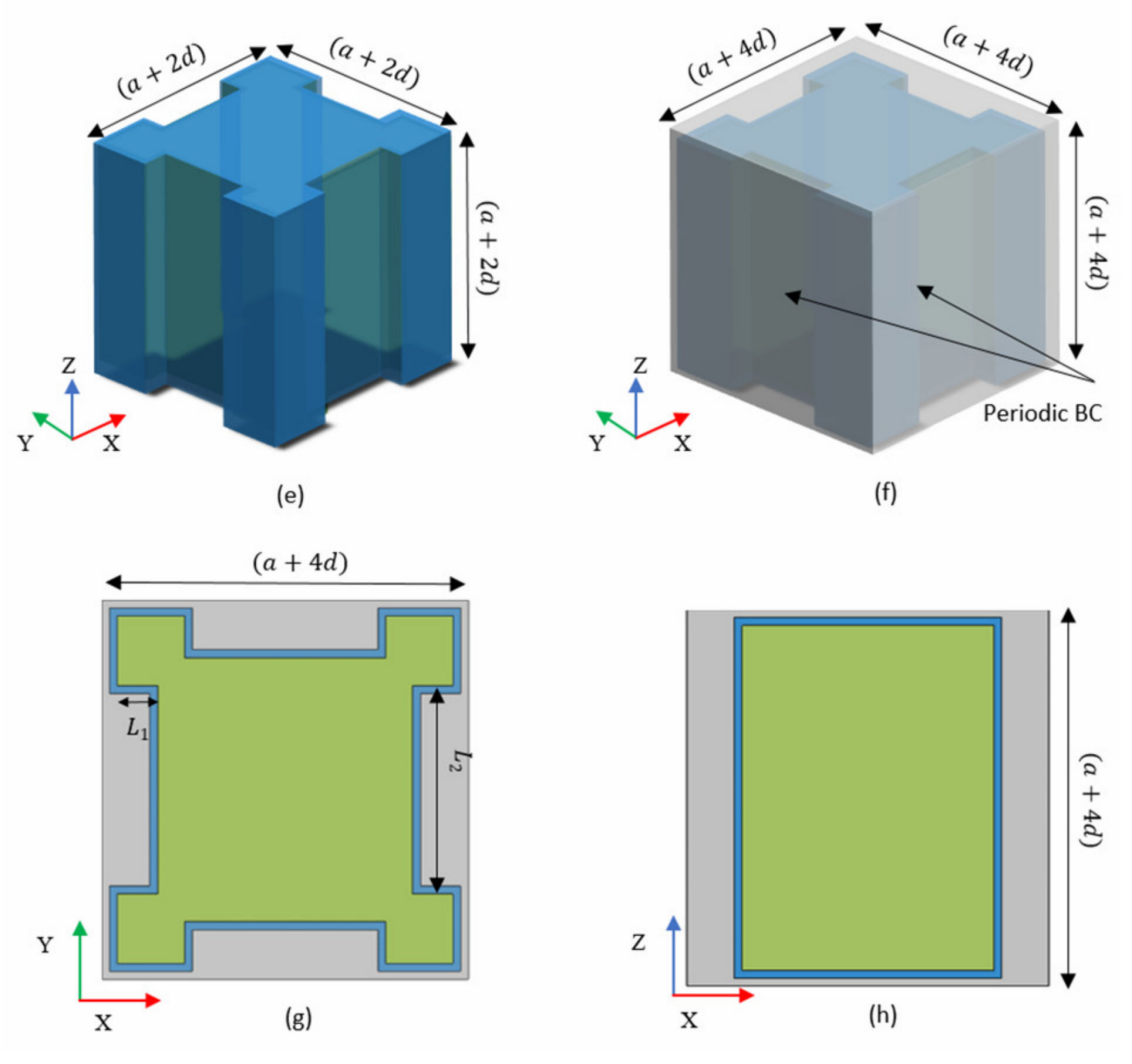
Detailed structure of the proposed metamaterial (**a**) Metamaterial array; (**b**) Unit arrangement; (**c**) Unit cell; (**d**) Steel scatter; (**e**) Rubber inclusion (including steel scatter inside); (**f**) Soil matrix (including steel scatter and rubber inclusion inside); (**g**) Middle cross section on x-y plane (**h**) Middle cross section on x-z plane.

The detailed structure of the proposed metamaterial is shown in Figure 1. The structure of the metamaterial is a square lattice structure on the x-y plane with a fixed lattice constant (Figure 1b). The metamaterial unit is composed of steel scatter, rubber inclusions, and soil matrix from inside to outside as shown in Figure 1c. The steel scatter is formed by removing four cuboids from a cube with side length ‘a’ and the size of the cuboids is ($L_{1}\times L_{2}\times a$) (Figure 1d). The rubber inclusion is coated on the surface of the steel scatter with uniform thickness ‘d’ (Figure 1e). The soil matrix is a cube with side length of ‘a+4d’ except for the internal steel block and rubber inclusion. The periodic Bloch boundary conditions are applied on the soil matrix along x and y directions (Figure 1f).

Here, we give a dispersion analysis process for finite element analysis to obtain the dispersion curve between frequency ω and wave vector ***k***. We assume that the three components of the metamaterial are isotropic and linear elastic materials. According to the elastic dynamic theory, the elastic motion equation with respect to the displacement is established [22,46].

(1) $$\rho \frac{\partial^{2}u_{i}}{\partial t^{2}}=\rho f_{i}+\left(\lambda +\mu \right)\frac{\partial^{2}u_{j}}{\partial x_{j}\partial x_{i}}+\mu \frac{\partial^{2}u_{i}}{\partial x_{j}\partial x_{j}}$$

where *λ* and μ are the Lamé constant, *ρ* is the mass density, $f_{i}$ is the body force, t is the time, $u_{i}$ is the displacement, $x_{i}$ is the position coordinates. i,j=1,2,3.

**Figure 2:**
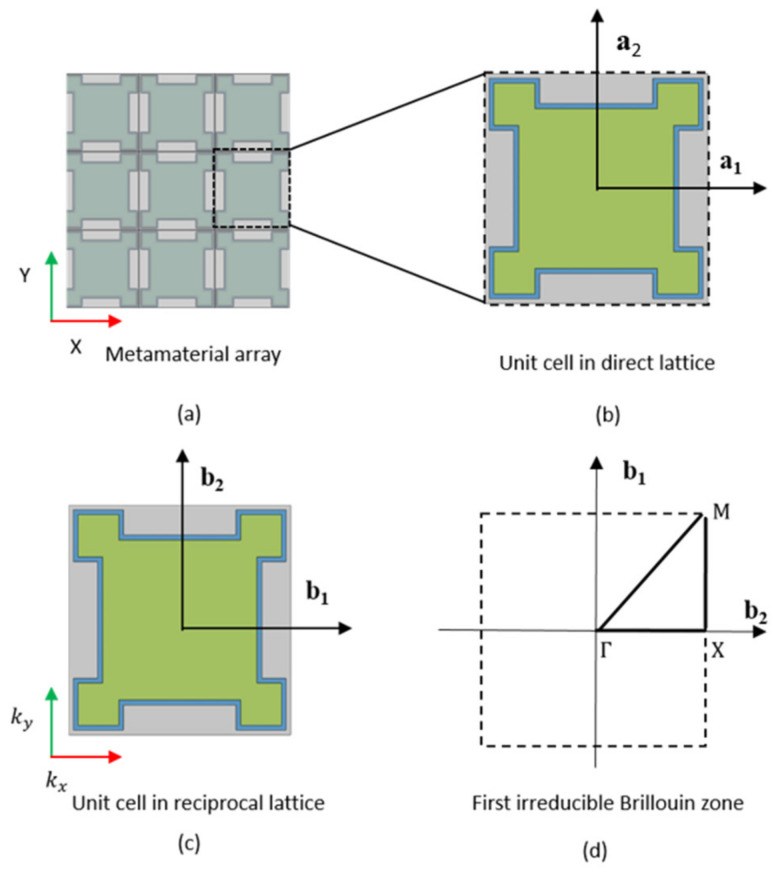
(**a**) Metamaterial array; (**b**) The direct lattice structure; (**c**) The reciprocal lattice structure; (**d**) The first irreducible Brillouin zone of the unit cell in the reciprocal lattice space.

For the square lattice structure of phononic crystal, the displacement solution $u\left(X,t\right)$ in Equation (1) can be described by the Floquet–Bloch theory [47], where the position vector is $X={\left[x_{1},x_{2}\right]}^{T}$. Figure 2b is the direct lattice of the structure unit, where the direct lattice base vector is $\left(a_{1},a_{2}\right)$. The corresponding translation vector is $T=n_{1}a_{1}+n_{2}a_{2}$ with $n_{1},n_{2}$ as positive integers. We set $X_{0}$ as the coordinate of the metamaterial reference unit. The position of any unit p in the lattice structure is

(2) $$X_{p}=X_{0}+T=X_{0}+n_{1}a_{1}+n_{2}a_{2}$$

where p=1,2,3… is the structure unit number. Figure 2c is the reciprocal lattice of the structure unit and the base vector is $\left(b_{1},b_{2}\right)$. The wave vector k is defined as the translation vector in reciprocal lattice space, which is expressed as $k=k_{x}b_{1}+k_{y}b_{2}$ with $k_{x},k_{y}$ as positive integers [46]. The direct lattice basis and reciprocal lattice basis should satisfy the following relationship $b_{i}\cdot a_{j}=2\pi \delta_{ij}$, where $\delta_{ij}$ is the Kronecker delta, i.e., $\delta_{ij}=1$ if i=j and $\delta_{ij}=0$ if i≠j. In our study, elastic wave field can be expressed as

(3) $$\psi \left(r,t\right)=\psi_{k}\left(r\right)e^{-i\omega t}$$

where $i=\sqrt{-1}$ is the imaginary number, ω is the angle frequency, and r is the coordinate vector [48]. According to the Bloch theory [49], the elastic wave field $\psi_{k}\left(r\right)$ in square lattice can be expressed as

(4) $$\psi_{k}\left(r\right)=\tilde{\psi_{k}}\left(r\right)e^{ik\cdot r}$$

where $e^{ik\cdot r}$ represents the plane wave. $\tilde{\psi_{k}}\left(r\right)$ is the periodic function of lattice, which satisfies $\tilde{\psi_{k}}\left(r\right)=\tilde{\psi_{k}}\left(r+T\right)$. Utilizing Equations (3) and (4), the displacement field $u\left(X_{0},t\right)$ can be expressed as

(5) $$u\left(X_{0},t\right)=u_{k}\left(X_{0}\right)e^{-i\omega t}={\tilde{u}}_{k}\left(X_{0}\right)e^{i\left(k\cdot X_{0}-\omega t\right)}$$

where ${\tilde{u}}_{k}\left(X_{0}\right)$ is the magnitude of the Bloch displacement field at the position $X_{0}$, ${\tilde{u}}_{k}\left(X_{0}\right)$ should satisfy the translation invariance in lattice structure, i.e.,

(6) $${\tilde{u}}_{k}\left(X_{0}\right)={\tilde{u}}_{k}\left(X_{0}+T\right)$$

Substituting Equation (6) into Equation (5), the displacement field at the position $X_{p}$ can be expressed as

(7) $$u\left(X_{p},t\right)=u\left(X_{0}+T,t\right)={\tilde{u}}_{k}\left(X_{0}+T\right)e^{i\left(k\cdot (X_{0}+T\right)-\omega t)}\;={\tilde{u}}_{k}\left(X_{0}\right)e^{i\left(k\cdot X_{0}-\omega t\right)}e^{ik\cdot T}=u\left(X_{0},t\right)e^{ik\cdot T}$$

Equation (7) represents the periodic displacement boundary condition of the metamaterial array. Considering the translation invariance in lattice structure, the change of wave amplitude is independent of the position of the unit cell. Hence, in order to obtain the dispersion relation between frequency ω and wave vector ***k***, we only need to consider its unit cell [50].

In order to obtain the dispersion relation, we use the finite element software COMSOL to calculate the eigenvalue Equation (8) [51,52,53].

(8) $$\left(K-\omega^{2}M\right)\cdot u=0$$

where K and M are total stiffness matrix and mass matrix of the unit, respectively. The dispersion analysis is conducted by taking the wave vector $k\left(k_{x},k_{y}\right)$ along the first irreducible Brillouin zone to calculate the eigenvalue of Equation (8). For the square lattice, the edge of irreducible Brillouin zone is the triangle ΓXM in the reciprocal lattice space (Figure 2d). The vertex coordinate is $\Gamma =0,0,X=\left(\frac{\pi }{c},0\right),\;M=\left(0,\frac{\pi }{C}\right)$, where c=a+4d is the lattice constant [54,55]. For a given real value of wave vector k, the frequency ω is the eigenvalue of Equation (8). The band gap characteristic can be obtained by analyzing the dispersion relationship between wave vector k and frequency ω [56].

Since the dispersion curve is calculated in an infinite array of phononic crystal, and the real structure scale in practical condition is usually finite, the band gap characteristics cannot fully reveal the shielding effects of the metamaterial to elastic waves. Therefore, we further carry out the frequency response analysis of the metamaterial structure in the finite medium to verify its attenuation effect for low frequency vibration in the next section.

## 3. Band Gap and Frequency Response Analysis

In this section, we obtain the band gap characteristics of the metamaterial through the FEM and explain the generation of the band gap. Based on the obtained band gap, we further carry out the frequency response analysis of the metamaterial structure. The work of this section is carried out by the finite element software COMSOL.

### 3.1. Band Structure and Band Gap

In order to obtain the dispersion relationship of the proposed metamaterial, we use FEM to conduct the modal analysis and plot the band structure diagram.

The process of the finite element analysis is followed.

(a) Finite element model and material properties

The unit cell of the proposed metamaterial is adopted as the finite element model as shown in Figure 1c. The geometric parameters of each component in a unit cell can be read from the Figure 1d–h. The side length of soil matrix is a+4d=2.5+4×0.05=2.7 m. The thickness of rubber d is 0.05 m. The notch sizes are $L_{1}=0.75\;m$, $L_{2}=0.3\;m$. The basic material properties of metamaterials are shown in Table 1.

(b) Boundary conditions

For the modal analysis of metamaterial structures, the Floquet–Bloch periodic boundary conditions are applied on the model along the x and y directions to calculate the characteristic frequency under different wave vectors ***k***.

(c) The boundary of the first irreducible Brillouin Zone

To obtain the dispersion relationship between k and ω, The wave vector k is swept along the boundary of the first irreducible Brillouin Zone (Γ-X, X-M, M-Γ) as shown in Figure 2d.

(d) The band structure diagram

**Figure 3:**
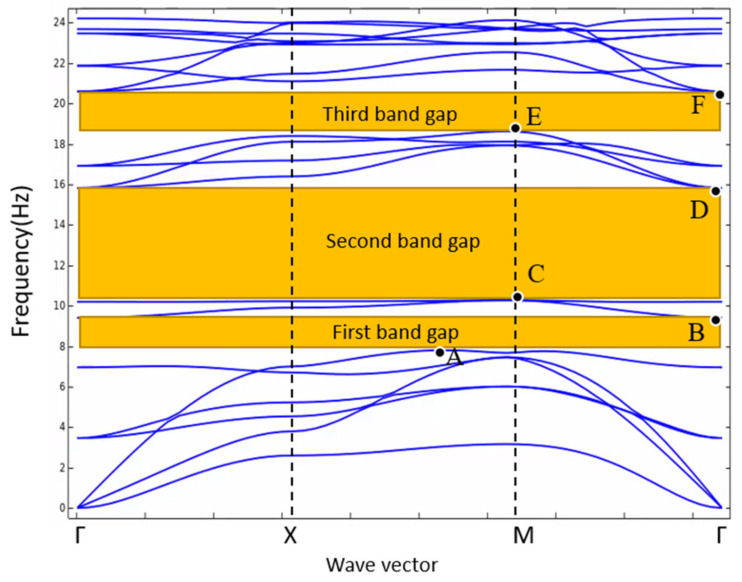
Band structure of the proposed metamaterial, the yellow bands represent three omnidirectional band gaps. A, B, C, D, E, and F are six boundary points of three band gaps.

For every wave vector k, there are infinite eigenvalues, i.e., the values of characteristic frequencies. Taking the values of characteristic frequencies below 25 Hz and the band structure can be plotted in the range of 0–25 Hz as shown in Figure 3.

From Figure 3, The range of frequency without the dispersion curves along Γ-X-M-Γ (all values of wave vector ***k***) is called the omnidirectional band gap. There are three obvious omnidirectional band gaps. The width of the first band gap is 1.603 Hz, ranging from 7.799 Hz to 9.402 Hz. The width of the second band gap is 5.543 Hz, ranging from 10.272 Hz to 15.815 Hz. The width of the third band gap is 1.968 Hz, ranging from 18.622 Hz to 20.590 Hz. It is worth noting that the total width of the omnidirectional band gaps below 20 Hz reaches 8.524 Hz, accounting for 42.6% of the range of 0–20 Hz, which means that the proposed metamaterial structure can block the elastic waves propagation in a fairly wide frequency range within 0–20 Hz. In addition, the band gap characteristics of metamaterials can be optimized by changing the geometric parameters and material parameters of unit cell, and the effect of relevant parameters will be discussed in Section 5.

### 3.2. Vibration Mode Analysis

The generation of the band gap can be explained according to the coupling effect between the vibration mode of the structure unit cell and the elastic wave propagation [57,58,59,60,61,62,63]. In this section, we analyze the vibration modes of the unit cell in boundary frequencies of band gaps to explain the starting and termination of each band gap.

**Figure 4:**
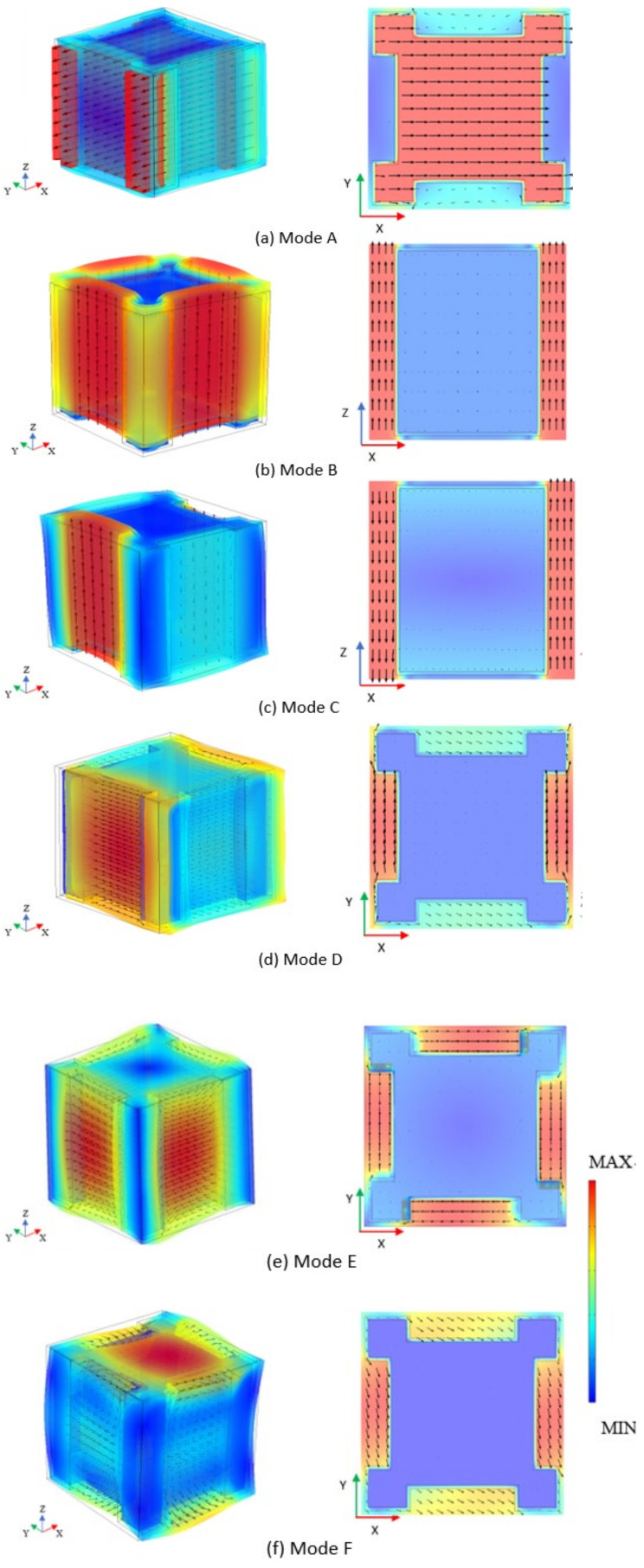
(**a**) Vibration mode of point A (main view and middle cross section on x-y plane; (**b**). Vibration modes of point B (main view and middle cross section on x-z plane; (**c**). Vibration modes of point C (main view and middle cross section on x-z plane; (**d**). Vibration modes of point D (main view and middle cross section on x-y plane; (**e**). Vibration modes of point E (main view and middle cross section on x-y plane; (**f**). Vibration modes of point F (main view and middle cross section on x-y plane.

There are three omnidirectional band gaps in the band structure from Figure 3. Each band gap has two boundary points at the starting frequency and the cut-off frequency, respectively. There are six band gap boundary points A, B, C, D, E, and F in the band structure diagram. Figure 4 is the vibration mode of metamaterial structure unit at six band gap boundary points (the main views and the middle cross sections on x-z plane or x-y plane), which are presented by the displacement field of the unit cell. The direction and length of the arrow in the diagram represent the direction and magnitude of the displacement at the starting position of this arrow.

Mode A and mode B are the vibration mode at the starting frequency and cut-off frequency of the first band gap, respectively. From the mode A, the steel scatter horizontally vibrates along the x-direction, and the soil matrix has slight vibration along the opposite direction. The vibration mode of the metamaterial unit cell shows the local resonance mode of the steel scatter. Because of the large mass of the steel scatter, its resonance frequency is low. When the frequency of elastic wave propagation is close to the resonance frequency of the steel block, the steel block will generate resonance and strongly couple with the plane mode of elastic wave propagation, localizing most of the energy of the elastic wave. Therefore, the elastic wave stops propagating, and the band gap starts. From the vibration mode of point B, the rubber vibrates longitudinally with the soil matrix, and the steel scatter has a reverse slight vibration. The vibration mode is shown as the local resonance of the soil matrix. Because of the low mass of the soil matrix, the coupling effect between the vibration mode of unit cell and the longitudinal shear mode of the elastic wave propagation is weak. Most of the energy of the elastic wave cannot be localized. The adjacent metamaterial units vibrate on the same phase and the elastic wave can continue to propagate. Hence the first band gap terminates. Mode C and mode D are the vibration mode at the starting frequency and the cut-off frequency of the second band gap, respectively. From the vibration mode at point C, the soil matrix shows longitudinal shear vibration mode. The vibration directions on both sides of the soil matrix are opposite, and the displacement amplitudes are the same. Shear effects on both sides of steel scatter offset each other and the total displacement of the unit cell is 0, which lead to the total displacement of the metamaterial structure to be 0. The elastic wave cannot propagate and the band gap starts. From the vibration mode of point D, the soil matrix and rubber inclusion locally vibrate along the y-direction, whose displacement along the x-direction is relatively small and the displacement of steel block is mostly zero. In other words, the total displacement amplitude of the metamaterial element is small. Furthermore, the mass of the soil matrix is small which causes the weak coupling between the vibration mode of metamaterial unit and the plane mode of elastic wave propagation. Therefore, most energy of elastic wave is retained and the elastic wave can continue to propagate. Hence the band gap terminates at mode D. Mode E and mode F are the vibration mode of the starting frequency and cut-off frequency of the third band gap, respectively. In the vibration mode of point E, the soil matrix and rubber inclusion are shown as torsional vibration mode, which has torque on the steel scatter. This vibration mode does not lead to the horizontal movement of the metamaterial structure unit. The total displacement of the metamaterial structure is 0, which means the elastic wave cannot cause the horizontal vibration of the unit cell. The band gap starts at mode E. When the frequency of elastic wave propagation increases to the frequency of mode F, the soil matrix horizontally vibrates on the x-y plane. The low mass of the soil matrix causes the weak coupling effect with the plane wave of the elastic wave. The elastic wave can continue to propagate and the band gap terminates.

For the proposed metamaterial structure, it can be found that when the frequency is low, the band gap mainly depends on the resonance frequency of the steel scatter. When the frequency is high, the band gap is related to the vibration mode of the soil matrix. When the total displacement of the unit cell on the x-y plane is 0, the band gap is generated; when the unit cell generates translation on the x-y plane, the band gap terminates.

### 3.3. Frequency Response Analysis

**Figure 5:**
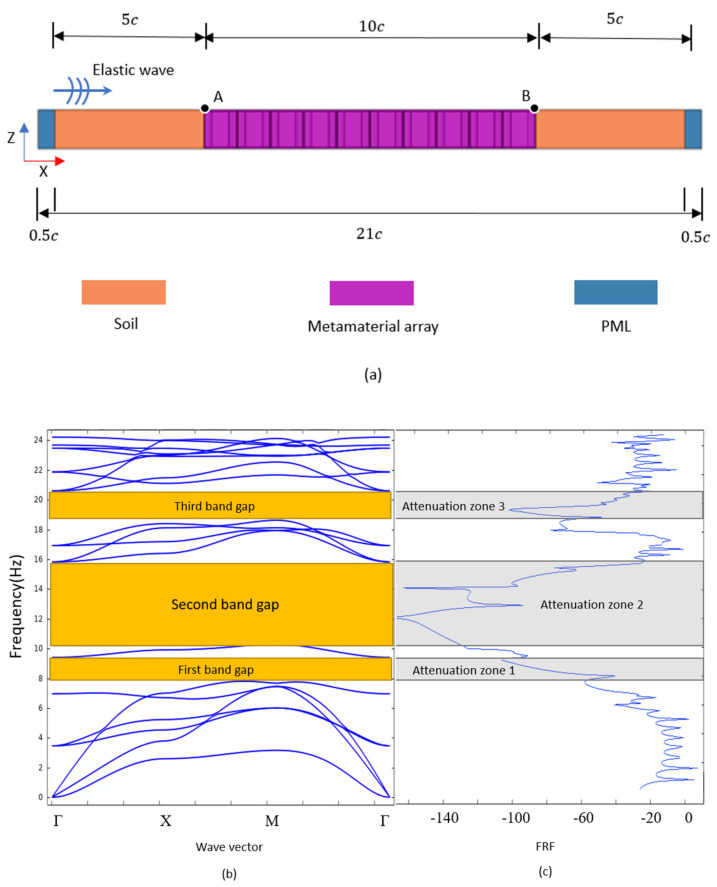
(**a**) Diagram of finite length model for the frequency response analysis; (**b**) Band structure diagram of the proposed metamaterial; (**c**) Frequency response curve of the metamaterial.

In order to verify the vibration attenuation on low frequency elastic waves of the proposed metamaterial structure, the frequency response of the finite array of the metamaterial is studied. In this section, we construct a FEM model of the frequency response analysis as shown in Figure 5a. This model is composed of metamaterial array, perfect matching layer (PML), and soil. Ten metamaterial units are arranged along the x-direction according to the periodic constant ‘c’. Soil material with length of 5c is placed on the left and right sides of the metamaterial array. In addition, the perfect matching layer (PML) with a length of 0.5c along the x-direction is added to the left and right ends of the soil material to absorb redundant interfering waves. This additional area will not produce any reflection of elastic waves. The material parameters of soil and metamaterial are the same as the Table 1. The material parameters of PML are the same as the soil. The plane wave with 100 MPa pressure amplitude is applied on the left side as the external excitation which propagates along the positive x-direction. Floquet–Bloch periodic boundary conditions along the y-direction are applied on the study model to achieve the infinite plane wave sources and realize the structure periodicity.

For a finite scale metamaterial array, the vibration attenuation effect is evaluated by the frequency response factor (FRF) which is defined as the ratio between response displacement $\mu_{1}$ and excitation displacement $\mu_{0}$, i.e., $\mathrm{FRF}=20\log\left(\frac{\mu_{1}}{\mu_{0}}\right)$ [37,64]. Here, $\mu_{1}$ is the maximum displacement at the ending point of the metamaterial array (point B), and $\mu_{0}$ is the maximum displacement at the starting point of the metamaterial array (point A). The frequency response analysis of elastic wave propagation in the range of 0–25 Hz is carried out with the step size of 0.1 Hz. The relationship between FRF and the excitation frequency of plane wave is shown in Figure 5c. From Figure 5c, with the increasing of transmission frequency, the FRF has three obvious attenuation sections. The frequency ranges of these three attenuation zones well agree with the three omnidirectional band gaps from the band structure diagram (Figure 5b). It is worth mentioning that the FRF has a significant upward trend between 16 and 18.6 Hz, which is very close to the pass band frequency range between the second band gap and the third band gap from the band structure diagram. Therefore, the frequency response analysis demonstrates that the proposed metamaterial structure can effectively attenuate the structural vibration caused by low frequency elastic waves.

## 4. Equivalent Mass–Spring Model

In the previous sections, we investigate the band gap characteristic based on FEM. However, FEM simulation usually cost much time. Here, we simplify the vibration mode of boundary frequency of band gap into an equivalent mass–spring model and theoretically calculate the band gap frequency range of the metamaterial structure.

### 4.1. Equivalent Model of Band Gap Boundary Vibration Modes

**Figure 6:**
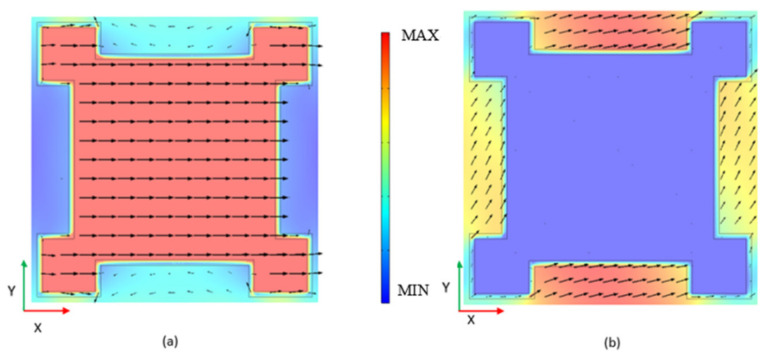

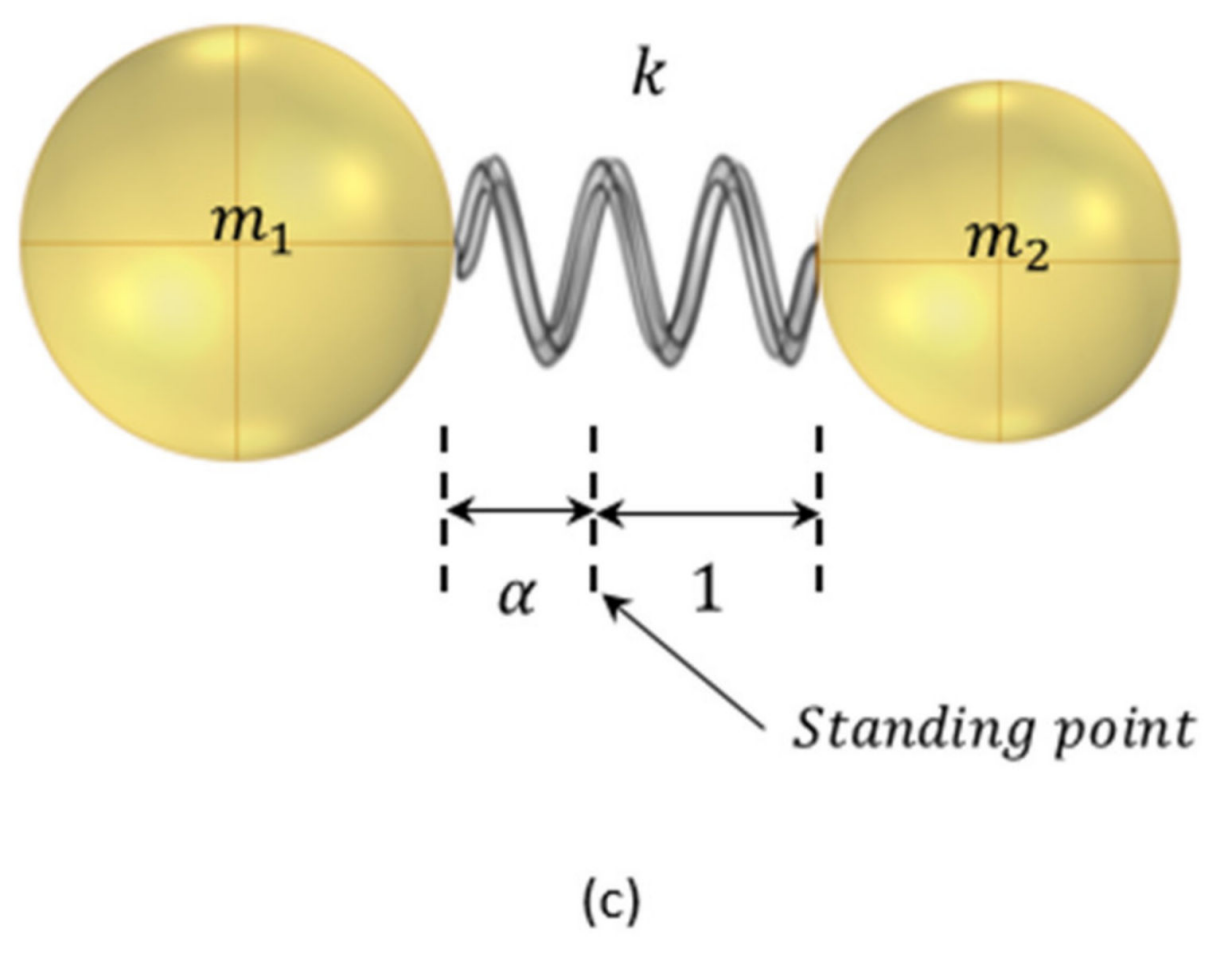
(**a**) The x-y middle cross section of unit cell vibration mode at starting frequency of band gap; (**b**) The x-y middle cross section of the unit cell vibration mode at cut-off frequency of band gap; (**c**) The equivalent model that can be used to describe these two vibration modes.

From Figure 6a, it can be found that the displacement of the steel scatter is along the positive x-direction and the rubber inclusion moves slightly. The soil matrix shows small displacement along the negative x-direction. From Figure 6b, it can be found that has the displacement of the soil matrix is along the positive x-direction, and the rubber inclusion moves slightly. The displacement of steel scatter is very small. These two vibration modes are shown as the relative motion of steel scatter and soil matrix along the x-direction. Since the width of rubber is very small, the rubber moves followed by the soil matrix or steel block scatter. Thus, the rubber inclusion can be regarded as a spring connecting the steel scatter and soil matrix. These two vibration modes in Figure 6 can be described uniformly by a mass–spring–mass model (as shown in Figure 6c). In this model, $m_{1}$ and $m_{2}$ are the equivalent masses and *k* is the equivalent stiffness of the spring. By solving the characteristic frequencies of the equivalent model under corresponding vibration modes, the start frequency and cut-off frequency of the band gap can be obtained. Next, we will determine the value of $m_{1},\;m_{2}$ and *k* to calculate the band gap frequency range.

### 4.2. Determination of Equivalent Mass and Equivalent Stiffness

(i) Equivalent mass $m_{1},m_{2}$

**Figure 7:**
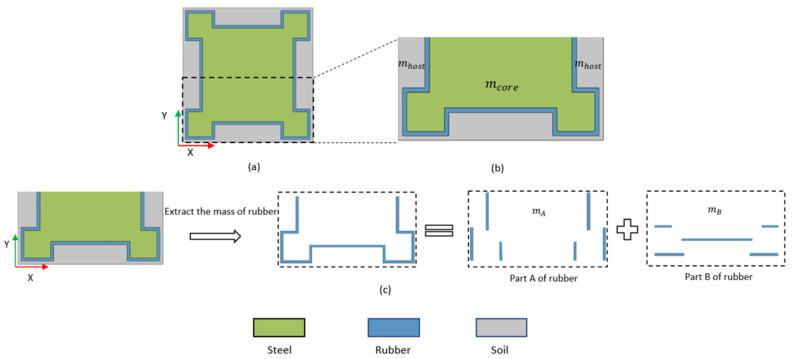
(**a**) Middle cross section of unit cell on x-y plane; (**b**) A half of the middle cross section of the unit cell on x-y plane, $m_{core}$ and $m_{host}$ is the mass of steel scatter and soil matrix, respectively; (**c**). The process of dividing rubber into part A and part B.

From Figure 6a,b, it can be found that the vibration modes of the unit cell are symmetrical about the x-axis. Because the structure of the unit cell is also symmetrical about the x-axis as shown in Figure 7a, only a half of the unit cell need to be considered as shown in Figure 7b. Since the mass of rubber cannot be ignored compared with soil and steel, the mass of rubber is needed to redistribute to the equivalent mass $m_{1}$ and $m_{2}$. As shown in Figure 7c, the mass of rubber can be divided into $m_{A}$ and $m_{B}$. Part A of rubber can be represented as a spring connecting the steel and soil because the stiffness of spring is mainly provided by the tension/compression deformation of this part when the soil and steel move relatively. Part B of rubber can be represented as the soil, because the stiffness provided by the deformation of this part is negligible and its displacement is very close to the soil. Next, the mass of part A is distributed to $m_{1}$ and $m_{2}$. α is defined as the mass ratio distributed into $m_{1}$ to $m_{2}$ from $m_{A}$, which is represents by the distances from standing point to $m_{1}$ and $m_{2}$ as shown in Figure 6c. In order to ensure that the mass of $m_{1}$ and $m_{2}$ is fixed when the spring vibrates, the position of the stand point should be fixed. According to the motion equation of mass–spring–mass model, the value of α should be $\alpha =\frac{m_{2}}{m_{1}}$.

**Figure 8:**
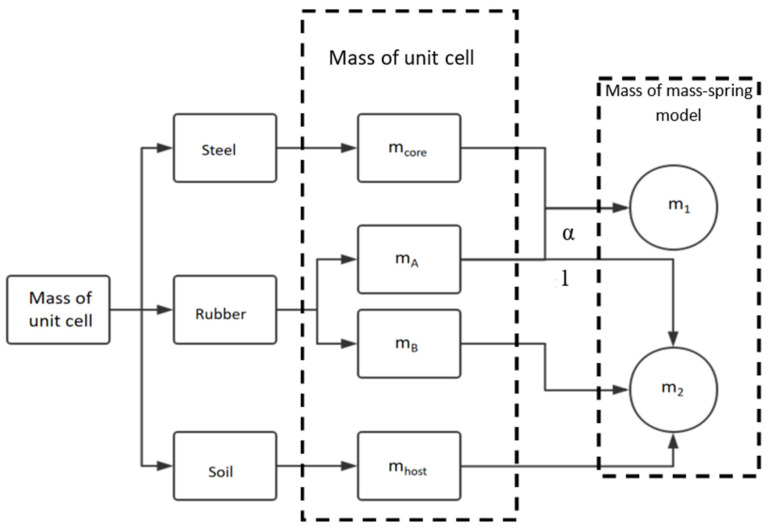
Mass corresponding relationship between structure unit cell and mass–spring–mass model.

Based on the above analysis, the mass distribution process between the structure unit cell and the mass–spring–mass model is shown in Figure 8.

Based on Figure 8, the calculation process of equivalent mass $m_{1}$ and $m_{2}$ is

(9) $$m_{1}=m_{core}+m_{A}\frac{\alpha }{1+\alpha }$$

(10) $$m_{2}=m_{host}+m_{B}+m_{A}\frac{1}{1+\alpha }$$

(11) $$\alpha =\frac{m_{2}}{m_{1}}.$$

Solving Equations (9)–(11), α can be calculated as

(12) $$\alpha =\frac{m_{A}+m_{B}+m_{host}}{m_{A}+m_{core}}.$$

From the Figure 7b, the rubber mass $m_{A}$ and $m_{B}$ are

(13) $$m_{A}=\rho_{rubber}d\left(a+2L_{2}-4d\right)$$

(14) $$m_{B}=\rho_{rubber}d\left(a+2L_{2}-2d\right).$$

The mass of steel scatter $m_{core}$ and soil matrix $m_{host}$ are

(15) $$m_{core}=\rho_{steel}(\frac{a^{2}}{2}-2L_{1}L_{2})$$

(16) $$m_{host}=\rho_{soil}(\frac{{\left(a+2d\right)}^{2}}{2}-\frac{{\left(a+2d\right)}^{2}}{2}+(2L_{1}-4d)L_{2})$$

where $\rho_{steel}$, $\rho_{soil}$ and $\rho_{rubber}$ represent the density of steel, soil, and rubber, respectively—i.e., $7850\frac{\mathrm{kg}}{m^{3}},\;1800\frac{\mathrm{kg}}{m^{3}},\mathrm{and}\;1300\;\mathrm{kg}/m^{3}$. The geometric parameters are the same as that in the finite element analysis: $L_{1}=0.75\;m$, $L_{2}=0.3\;m$, d=0.05 m, a=2.5 m.

Solving above equations, the value of $m_{1}$, $m_{2}$ and α are 17,489.0 kg, 2350.2 kg, and 0.1344, respectively.

(ii) Equivalent stiffness k

The rubber inclusion in part A can be regarded as many springs with length ‘d’ in parallel connection. The equivalent stiffness k of the spring is

(17) $$k=\frac{c_{1}\left(a-4d+2L_{2}\right)}{d}$$

(18) $$c_{1}=\lambda +2\mu$$

where λ and μ are the Lamé constant of rubber. Solving Equations (17) and (18), the equivalent stiffness can be calculated as 1.75 × 10^7^.

### 4.3. Bandgap Boundary Frequencies

**Figure 9:**
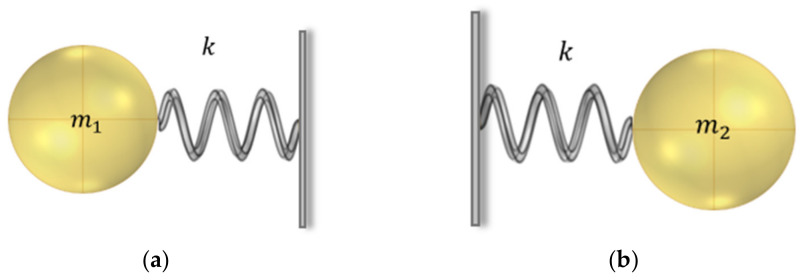
(**a**) Equivalent model of band gap at starting frequency. (**b**) Equivalent model of band gap at cut-off frequency.

The vibration mode of the band gap at starting frequency (as shown in Figure 6a) is mainly shown as the horizontal displacement of the steel scatter and the slight displacement of the rubber inclusion. Because of the small displacement of soil matrix, only the vibration of steel scatter and the rubber part A are needed to be considered. Therefore, the equivalent mass $m_{2}$ in the mass–spring–mass model can be further considered as a rigid wall with the displacement of 0. The equivalent model at the starting frequency of the band gap can be further simplified to the single mass–spring model shown in Figure 9a.

The characteristic frequency of the model is

(19) $$f_{1}=\sqrt{\frac{k}{m_{1}}}$$

The vibration mode of the band gap at cut-off frequency (as shown in Figure 6b) is mainly shown as the horizontal displacement of the soil matrix and the slight displacement of the rubber inclusion. Contrary to the vibration mode at the starting frequency, the displacement of the steel scatter is very small which can be represented as a rigid wall. Therefore, the equivalent model of band gap at cut-off frequency can be further simplified to the single mass–spring model shown in Figure 9b.

The characteristic frequency of the model is

(20) $$f_{2}=\sqrt{\frac{k}{m_{2}}}$$

According to the value of equivalent mass $m_{1},m_{2}$ and equivalent stiffness k, the starting frequency and cut-off frequency can be calculated as $f_{1}=7.555\;\mathrm{Hz}$, $f_{2}=20.610\;\mathrm{Hz}$.

The theoretical analysis range of band gap frequency is 7.555 Hz–20.610 Hz and the bang gap range of finite element analysis is 7.799–20.590 Hz. These results agree well. Therefore, the band gap frequency range of the metamaterial structure can be calculated faster through the equivalent mass–spring model. It also can be found from Equations (19) and (20) that the starting frequency and the cut-off frequency is very close to the resonance frequencies of steel scatter and soil matrix, respectively. This phenomenon agrees with the vibration mode analysis conclusion in Section 3.2.

## 5. Optimization of Band Gap Characteristics

The geometric parameters and material parameters of seismic metamaterial are important factors affecting the band gap characteristic [35,62,63,64,65,66,67]. In order to further study the effect of these factors on the band gap characteristics and obtain the band gap with lower frequency and larger bandwidth, we investigate the band gap characteristics using the FEM and equivalent mass–spring model under different elastic modulus of the rubber inclusion, different lattice constant of the metamaterial array and different filling ratio of the steel scatter, respectively.

### 5.1. Effect of Lattice Constants of Metamaterial Arrays

**Figure 10:**
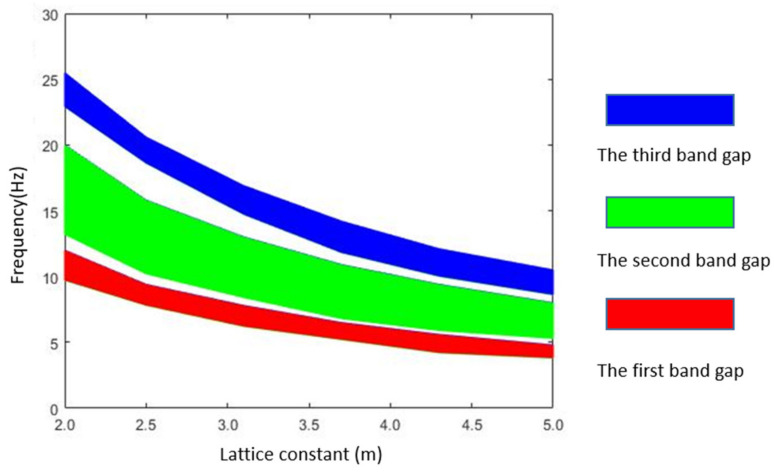
Effect of lattice constant on band gap distribution.

**Figure 11:**
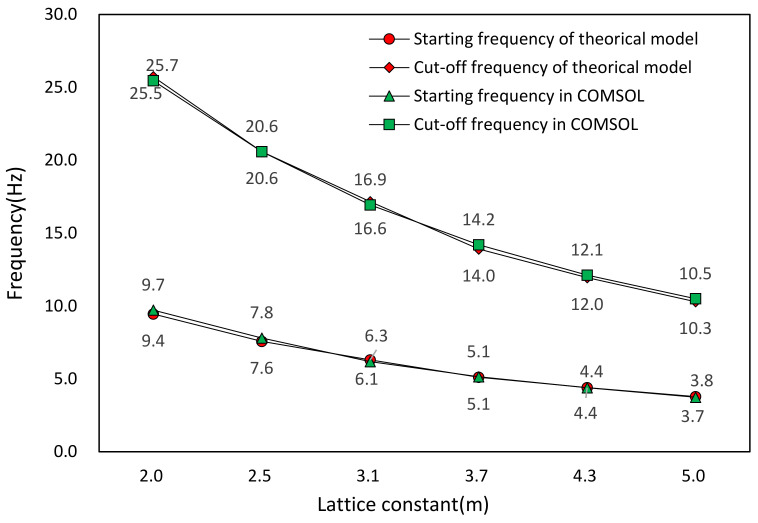
Theoretical and FEM results of band gap ranges under different lattice constants.

We first analyze the relation between the distribution of the band gap and the lattice constant of the metamaterial array by FEM. The lattice constants of the metamaterial unit are set as 2.5, 3.1, 3.7, 4.3, 5.0, and 5.6 m, respectively. For the different lattice constant ‘a’, the finite element simulation of the characteristic frequency of the metamaterial structure is carried out and the band gap frequency ranges are obtained. Table 2 shows the band gap characteristics under different lattice constants. The corresponding band gap distributions is shown in Figure 10. We also adopt the equivalent mass–spring model to calculate the starting frequency and the cut-off frequency of the band gaps. The theoretical analysis results are compared with the FEM results as shown in Figure 11.

From Figure 10, it can be found that the starting frequencies and cut-off frequencies of band gaps decrease with the increasing of lattice constants. Since the filling ratio of the scatter is unchanged, the mass of the scatter and the mass of the matrix will increase with the increasing of lattice constants and their resonance frequencies will decrease. Therefore, the starting frequency and the cut-off frequency become lower. Figure 11 shows the comparison between the band gap frequency results of the equivalent mass–spring model and the FEM, which agree well. It proves that it is reasonable to predict the band gap characteristics of the metamaterial structure by using the equivalent mass–spring model within a certain ranges of lattice constants.

### 5.2. Effect of Elastic Modulus of Rubber Inclusion

**Figure 12:**
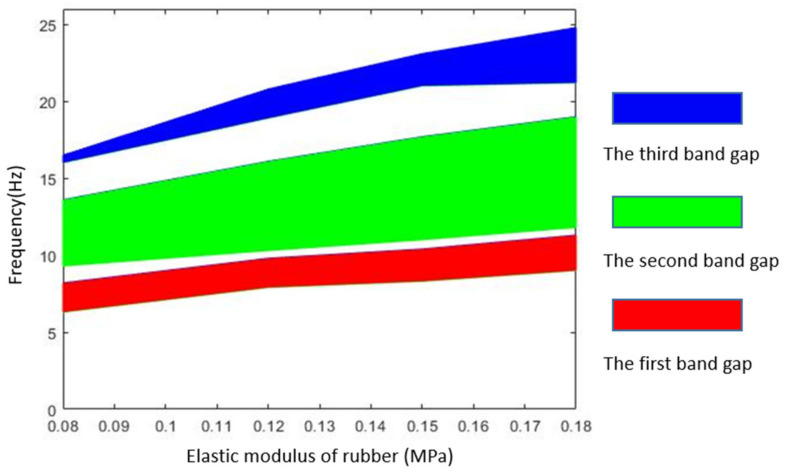
Effect of elastic modulus of rubber on band gap distribution.

**Figure 13:**
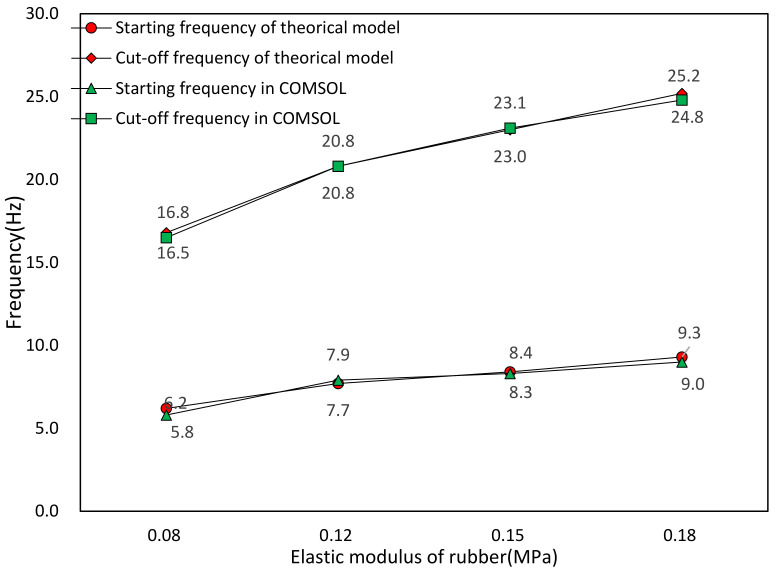
Theoretical and FEM results of band gap ranges under different elastic moduli of rubber.

Rubber inclusion is often used as spring in the band gap analysis of seismic metamaterial, so it is necessary to study the effect of elastic modulus on band gap characteristic. We analyze the effect of elastic modulus of rubber inclusion on the band gap distribution through FEM simulation and equivalent mass–spring model. Except for the different elastic modulus of rubber, the rest structural parameters and geometric parameters of metamaterial are the same. The elastic modulus of rubber is set as 0.08, 0.12, 0.15, and 0.18 MPa, respectively. Table 3 is the FEM results of band gap characteristics under different elastic modulus of rubber, where the relation between elastic modulus of rubber inclusion and band gap distribution is plotted in Figure 12. Figure 13 is the results comparison of band gap characteristic between the FEM and the equivalent mass–spring model.

From Figure 12, it can be found that the frequency of the band gap and the total bandwidth increase with increasing of the elastic modulus of the rubber. This phenomenon is due to the increasing equivalent stiffness of the spring which leads to the increasing resonance frequencies of the scatter and the matrix. When the elastic modulus of rubber exceeds 0.15 MPa, the band gap between almost covers the frequency range of 10–20 Hz. Therefore, increasing the elastic modulus of inclusions is an effective method to increase the bandwidth. However, with the increasing of bandwidth the starting frequency is also rising, which is not a favorable trend for the low frequency vibration attenuation. Researchers can adjust the elastic modulus of rubber appropriately according to the required band gap width and band gap frequency ranges. From Figure 13, the calculation results of band gap boundary frequencies by the FEM and the equivalent mass–spring model reach agreement, which indicates that in a certain range of rubber elastic modulus, the equivalent mass–spring model is applicable to calculate the band gap characteristics of the metamaterial.

### 5.3. Effect of Filling Ratio of Steel Scatter

**Figure 14:**
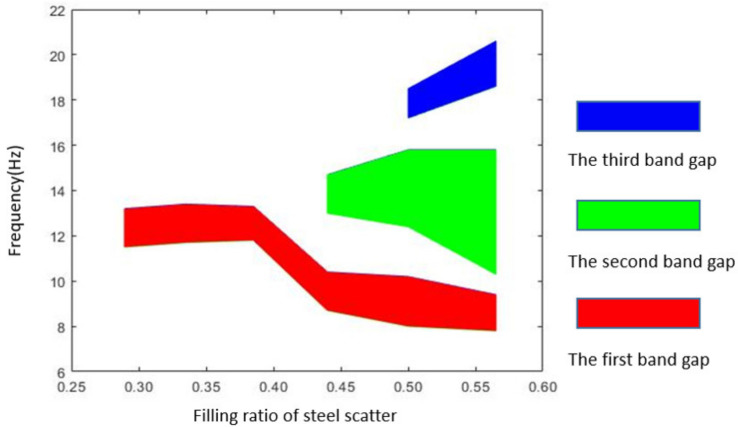
Effect of the filling ratio of steel scatter on the band gap distribution.

**Figure 15:**
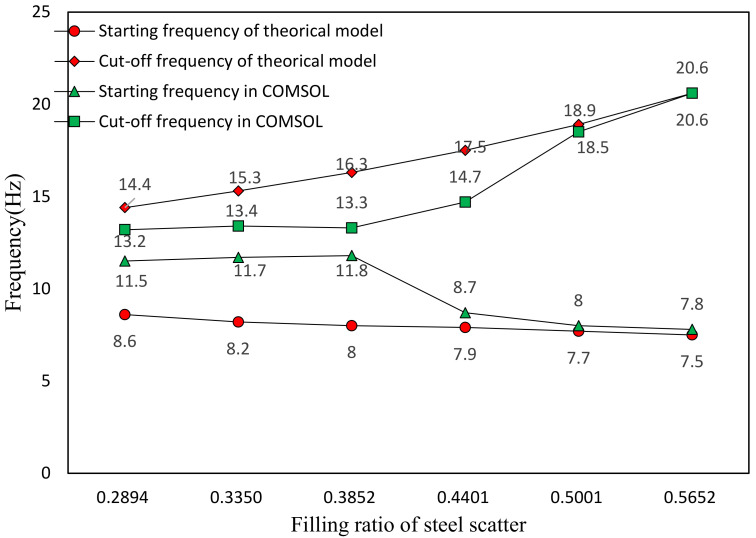
Theoretical and FEM results of band gap ranges under different filling ratio of scatter.

The band gap characteristics under different filling ratios of steel scatter are analyzed by FEM and equivalent mass–spring model respectively. Filling ratio is the volume ratio of scatter in seismic metamaterial. In our research, different filling ratio is realized by changing the side length ‘a’ in steel scatter unit model (Figure 2d), which is set as 2, 2.1, 2.2, 2.3, 2.4, and 2.5 m, respectively. The thickness of the rubber inclusion remains 0.05 m, and the lattice constant of the metamaterial remains 2.7 m. Thus, the corresponding filling ratios are 0.2894, 0.3350, 0.3852, 0.4401, 0.5001, and 0.5652, respectively. The relationship between the distribution of band gap and the filling ratio of steel scatter through FEM is shown in Figure 14. Table 4 illustrates the FEM results of the band gap distribution characteristics under different filling ratio of steel scatter. Figure 15 shows the comparison between the calculation results of the equivalent mass-model and the FEM on the band gap boundary frequency.

From Figure 14, it can be found when the filling ratio of the scatter is lower than 0.4, only one band gap can be formed, and the bandwidth of the first band gap is mostly unchanged. When the filling ratio rises to 0.44, the second band gap appears. Until the scatter filling ratio reaches 0.57, the third band gap is generated. In general, with increasing of the scatter filling ratio, the number of band gaps increases and the total bandwidth also increases. Figure 15 shows that when the filling ratio is small, the FEM results are greatly different from the results of the equivalent mass–spring model. With the increasing of the filling ratio, when the number of band gaps increases to three, the FEM results are almost consistent with the theoretical results. This phenomenon is due to the vibration mode of the metamaterial structure unit under low filling ratio cannot be accurately described as the equivalent mass–spring model. Therefore, we think that the equivalent mass–spring model is only applicable to describe the band gap characteristics with a certain range of scatter filling ratio, which is generally higher than 0.5. In addition, increasing the filling ratio of steel scatter will increase the total bandwidth but the starting frequency does not increase, which is good for the vibration attenuation under low frequency.

Based on the above analysis, in order to obtain band gap characteristics with lower frequency and larger bandwidth, researchers can optimize the band gap characteristics by increasing the elastic modulus of inclusion, increasing the filling ratio of the scatter or choosing proper lattice constant of the metamaterial unit.

## 6. Conclusions

In this study, we propose a ternary seismic metamaterial to attenuate structural vibration induced by low-frequency elastic waves. Through FEM analysis, the band gap characteristics are obtained, and the frequency response factor is calculated. Then an equivalent mass–spring model is proposed to calculate the theoretical band gap frequency range of the metamaterial structure. On this foundation, the optimization for the band gap characteristics is investigated by changing the geometric parameters and material parameters of the metamaterial components. According to the results and analysis, the following conclusion can be drawn:

1. The proposed metamaterial structure can generate an omnidirectional band gap of 8.5 Hz width in the low frequency range of 0–20 Hz and the low frequency vibration in the band gap range can be well attenuated.
2. The results of the proposed equivalent mass–spring model agree well with the FEM results. This model provides a convenient method to obtain the band gap range.
3. The band gap with lower frequency and larger bandwidth can be obtained by appropriately increasing the filling ratio of the scatter, the elastic modulus of the inclusion, or choosing the lattice constant of the metamaterial array.

This study can provide an important reference for the attenuation of low-frequency vibration of infrastructure such as nuclear power plants.

## Figures and Tables

**Table 1:** Mechanical properties of materials [40,41].

Material	Young’s Modulus E (GPa)	Poisson’s Ratio ν	Destiny *ρ* (kg/m^3^)
Steel	210	0.3	7850
Rubber	0.00012	0.468	1300
Soil	0.03	0.3	1800

**Table 2:** Distribution of band gap under different lattice constants.

Lattice Constant (m)	Number of Band Gaps	Band Gap Range (Hz)	Total Omnidirectional Band Gap Width (Hz)
2	3	9.7–12.0; 13.2–20.0; 22.9–25.5	11.7
2.5	3	7.8–9.4; 10.2–15.8; 18.6–20.6	9.2
3.1	3	6.2–7.8; 8.4–13.0; 14.7–16.9	8.4
3.7	3	5.2–6.5; 6.8–10.9; 11.8–14.2	7.6
4.3	3	4.2–5.6; 5.9–9.4; 10.0–12.1	7.0
5	3	3.8–4.8; 5.3–8.0; 8.6–10.5	5.6

**Table 3:** Band gap distribution under different rubber elastic modulus.

Elastic Modulus of Rubber (MPa)	Number of Band Gaps	Band Gap Range (Hz)	Total Omnidirectional Band Gap Width (Hz)
0.08	3	6.5–8.3; 9.3–13.6; 16.8–17.8	7.1
0.12	3	7.8–9.4; 10.2–15.8; 18.6–20.6	9.2
0.15	3	8.5–10.7; 11.0–17.6; 20.4–23.3	12.7
0.18	3	9.3–11.5; 11.6–19.0; 21.2–24.5	12.9

**Table 4:** Band gap distribution under different scatter filling rates.

Filling Rate	Number of Band Gaps	Band Gap Range (Hz)	Total Omnidirectional Band Gap Width (Hz)
0.2894	1	11.5–13.2	1.7
0.3350	1	11.7–13.4	1.7
0.3852	1	11.8–13.3	1.5
0.4401	2	8.7–10.4; 13.0–14.7	3.4
0.5001	3	8.0–10.2; 12.4–15.8; 17.2–18.5	6.9
0.5652	3	7.8–9.4; 10.2–15.8; 18.6–20.6	9.2

## Data Availability

The data presented in this study are available on request from the corresponding author.

## References

[1] Ellsworth W.L. (2013). Injection-induced earthquakes. Science.

[2] Brownjohn J.M.W., De Stefano A., Xu Y., Wenzel H., Aktan A.E. (2011). Vibration-based monitoring of civil infrastructure: Challenges and successes. J. Civ. Struct. Health Monit..

[3] Bajcar T., Cimerman F., Širok B., Ameršek M. (2012). Impact assessment of traffic-induced vibration on natural gas transmission pipeline. J. Loss Prev. Process Ind..

[4] Paulay T. (1986). The design of ductile reinforced concrete structural walls for earthquake resistance. Earthq. Spectra.

[5] Hajirasouliha I., Pilakoutas K., Moghaddam H. (2011). Topology optimization for the seismic design of truss-like structures. Comput. Struct..

[6] Xiang J., Shi Z.F., Wang S.J., Mo Y.L. (2012). Periodic materials-based vibration attenuation in layered foundations: Experimental validation. Smart Mater. Struct..

[7] Harvey P.S., Kelly K.C. (2016). A review of rolling-type seismic isolation: Historical development and future directions. Eng. Struct..

[8] Brûlé S., Enoch S., Guenneau S. (2020). Emergence of seismic metamaterials: Current state and future perspectives. Phys. Lett. A.

[9] Mu D., Shu H., Zhao L., An S. (2020). A review of research on seismic metamaterials. Adv. Eng. Mater..

[10] Lim C.W. (2021). From photonic crystals to seismic metamaterials: A review via phononic crystals and acoustic metamaterials. Arch. Comput. Methods Eng..

[11] Zhang H., Wen J., Xiao Y., Wang G., Wen X. (2015). Sound transmission loss of metamaterial thin plates with periodic subwavelength arrays of shunted piezoelectric patches. J. Sound Vib..

[12] Sukhovich A., Merheb B., Muralidharan K., Vasseur J.O., Pennec Y., Deymier P.A., Page J.H. (2009). Experimental and theoretical evidence for subwavelength imaging in phononic crystals. Phys. Rev. Lett..

[13] Sun F., Xiao L. (2019). Bandgap characteristics and seismic applicationsof inerter-in-lattice metamaterials. J. Eng. Mech..

[14] Martinsson P., Movchan A. (2003). Vibrations of lattice structures and phononic band gaps. Q. J. Mech. Appl. Math..

[15] Valentine J., Zhang S., Zentgraf T., Ulin-Avila E., Genov D.A., Bartal G., Zhang X. (2008). Three-dimensional optical metamaterial with a negative refractive index. Nature.

[16] Sigalas M.M., Economou E.N. (1992). Elastic and acoustic wave band structure. J. Sound Vib..

[17] Kushwaha M.S., Halevi P., Dobrzynski L., Djafari-Rouhani B. (1993). Acoustic band structure of periodic elastic composites. Phys. Rev. Lett..

[18] Pyskir A., Collet M., Dimitrijevic Z., Lamarque C.H. (2021). Enhanced Vibration Isolation with Prestressed Resonant Auxetic Metamaterial. Materials.

[19] Sharmar A.K., Kosta M., Shmuel G., Amir O. (2022). Gradient-based topology optimization of soft dielectrics as tunable phononic crystals. Compos. Struct..

[20] Bortot E., Amir O., Shmuel G. (2018). Topology optimization of dielectric elastomers for wide tunable band gaps. Int. J. Solids Struct..

[21] Meng H., Chronopoulos D., Bailey N., Wang L. (2020). Investigation of 2D rainbow metamaterials for broadband vibration attenuation. Materials.

[22] Varma T.V., Ungureanu B., Sarkar S., Craster R., Guenneau S., Brûlé S. (2021). The influence of clamping, structure geometry, and material on seismic metamaterial performance. Front. Mater..

[23] Zeng Y., Xu Y., Deng K.K., Peng P., Yang H.W., Muzamil M., Du Q. (2019). A broadband seismic metamaterial plate with simple structure and easy realization. J. Appl. Phys..

[24] Muhammad L.C.W., Reddy J.N. (2019). Built-up structural steel sections as seismic metamaterials for surface wave attenuation with low frequency wide bandgap in layered soil medium. Eng. Struct..

[25] Liu Z.Y., Zhang X.X., Mao Y.W., Zhu Y.Y., Yang Z.Y., Chan C.T., Sheng P. (2003). Locally resonant sonic materials. Phys. B Condens. Matter.

[26] Chen Y., Wang L. (2014). Isolation of surface wave-induced vibration using periodically modulated Piles. Int. J. Appl. Mech..

[27] Ungureanu B., Achaoui Y., Enoch S., Brûlé S., Guenneau S. (2015). Auxetic-like metamaterials as novel earthquake protections. EPJ Appl. Metamater..

[28] Huang K., Shui G., Wang Y., Wang Y. (2020). Meta-arrest of a fast propagating crack in elastic wave metamaterials with local resonators. Machanics Mater..

[29] Sadat S.M., Yang R.Y. (2020). A Machine learning based approach for phononic crystal property discovery. J. Appl. Phys..

[30] Brûlé S., Javelaud E.H., Enoch S., Guenneau S. (2014). Experiment on seismic metamaterials: Molding surface waves. Phys. Rev. Lett..

[31] Liu J.L., Feng G.S., Zhang J., Zhao X.Y., Yu C.Q., Zhao M. (2018). Vibration isolation mechanism of concrete piles for Rayleigh waves and sand foundations. Shock Vib..

[32] Colombi A., Roux P., Guenneau S., Gueguen P., Craster R.V. (2016). Forests as a natural seismic metamaterial: Rayleigh wave bandgaps induced by local resonances. Sci. Rep..

[33] Yan Y., Laskar A., Cheng Z., Meng F., Tang Y., Mo Y.L., Shi Z. (2014). Seismic isolation of two dimensional periodic foundations. J. Appl. Phys..

[34] Witarto W., Wang S.J., Yang C.Y., Nie X., Mo Y.L., Chang K.C., Tang Y., Kassawara R. (2018). Seismic isolation of small modular reactors using metamaterials. Am. Inst. Phys. Syst. Struct..

[35] Chen Y., Qian F., Scarpa F., Zuo L., Zhuang X. (2019). Harnessing multi-layered soil to design seismic metamaterials with ultralow frequency band gaps. Mater. Des..

[36] Guo Q., Yao W., Li W., Gupta N. (2021). Constitutive models for the structural analysis of composite materials for the finite element analysis: A review of recent practices. Compos. Struct..

[37] Khalid S., Lee J., Kim H.S. (2022). Series solution-based approach for the interlaminar stress analysis of smart composites under thermo-electro-mechanical loading. Mathematics.

[38] Lee H.S., Shin J.K., Msolli S., Kim H.S. (2019). Prediction of the dynamic equivalent stiffness for a rubber bushing using the finite element method and empirical modeling. Int. J. Mech. Mater. Design..

[39] Achaoui Y., Ungureanu B., Enoch S., Brûlé S., Guenneau S. (2016). Seismic waves damping with arrays of inertial resonators. Extrem. Mech. Lett..

[40] Achaoui Y., Antonakakis T., Brûlé S., Craster R.V., Enoch S., Guenneau S. (2017). Clamped seismic metamaterials: Ultra-low frequency stop bands. New J. Phys..

[41] Miniaci M., Krushynska A., Bosia F., Pugno N.M. (2016). Large scale mechanical metamaterials as seismic shields. New J. Phys..

[42] Krödel S., Thomé N., Daraio C. (2015). Wide band-gap seismic metastructures. Extrem. Mech. Lett..

[43] Zeng Y., Xu Y., Yang H., Muzamil M., Xu R., Deng K., Peng P., Du Q. (2020). A Matryoshka-like seismic metamaterial with wide band-gap characteristics. Int. J. Solids Struct..

[44] Xu Y., Xu R., Peng P., Yang H., Zeng Y., Du Q. (2019). Broadband H-shaped seismic metamaterial with a rubber coating. EPL Europhys. Lett..

[45] Colombi A., Colquitt D., Roux P., Guenneau S., Craster R.V. (2016). A seismic metamaterial: The resonant metawedge. Sci. Rep..

[46] Cheng Z.B., Shi Z.F. (2018). Composite periodic foundation and its application for seismic isolation. Earthq. Eng. Struct. Dyn..

[47] Phani A.S., Woodhouse J., Fleck N. (2006). Wave propagation in two-dimensional periodic lattices. J. Acoust. Soc. Am..

[48] Bertoldi K., Boyce M.C. (2008). Wave propagation and instabilities in monolithic and periodically structured elastomeric materials undergoing large deformations. Phys. Rev. B.

[49] Kittel C., Hellwarth R.W. (1976). Introduction to Solid State Physics.

[50] Guenneau S., Movchan A.B. (2004). Analysis of elastic band structures for oblique incidence. Arch. Ration. Mech. Anal..

[51] Hussin M.I. (2009). Reduced Bloch mode expansion for periodic media band structure calculations. Proc. R. Soc. A.

[52] Collet M., Ouisse M., Ruzzene M., Ichchou M.N. (2011). Floquet-Bloch decomposition for the computation of dispersion of two-dimensional periodic, damped mechanical systems. Int. J. Solids Struct..

[53] Palermo A., Marzani A. (2020). A reduced Bloch operator finite element method for fast calculation of elastic complex band structures. Int. J. Solids Struct..

[54] Xia B., Wang G., Zheng S. (2018). Robust edge states of planar phononic crystals beyond high-symmetry points of Brillouin zones. J. Mech. Phys. Solids.

[55] Maurin F., Claeys C., Deckers E., Desmet W. (2018). Probability that a band-gap extremum is located on the irreducible Brillouin-zone contour for the 17 different plane crystallographic lattices. Int. J. Solids Struct..

[56] Jarlebring E., Mele G., Runborg O. (2017). The waveguide eigenvalue problem and the tensor infinite Arnoldi method. Siam J. Sci. Comput..

[57] Dong Y., Yao H., Du J., Zhao J., Jiang J. (2017). Research on local resonance and Bragg scattering coexistence in phononic crystal. Mod. Phys. Lett. B.

[58] Zhang Z., Han X.K. (2016). A new hybrid phononic crystal in low frequencies. Phys. Lett. A.

[59] Sainidou R., Stefanou N., Modinos A. (2002). Formation of absolute frequency gaps in three-dimensional solid phononic crystals. Phys. Rev. B.

[60] Song A., Wang X., Chen T., Jiang P., Bao K. (2016). Low-frequency bandgaps of two-dimensional phononic crystal plate composed of asymmetric double-sided cylinder stubs. Int. J. Mod. Phys. B.

[61] Coffy E., Lavergne T., Addouche M., Euphrasie S., Vairac P., Khelif A. (2015). Ultra-wide acoustic band gaps in pillar-based phononic crystal strips. J. Appl. Phys..

[62] Zhao H., Liu Y., Wang G., Wen J., Yu D., Han X., Wen X. (2005). Resonance modes and gap formation in a two-dimensional solid phononic crystal. Phys. Rev. B.

[63] Kafesaki M., Sigalas M.M., Economou E.N. (1995). Elastic wave band gaps in 3-D periodic polymer matrix composites. Solid State Commun..

[64] Lim C.W. (2019). Elastic waves propagation in thin plate metamaterials and evidence of low frequency pseudo and local resonance bandgaps. Phys. Lett. A.

[65] Bilal O.R., Hussein M.I. (2013). Tramploine metamaterials: Local resonance enhancenment by springboards. Appl. Phys. Lett..

[66] Chen L., Bian Y.-S., Zhou R. (2019). Large band gaps of petal-shaped acoustic metamaterials based on local resonance. J. Vib. Eng. Technol..

[67] Wang C., Yao X., Wu G., Tang L. (2021). Complete vibration band gap characteristics of two-dimensional periodic grid structures. Compos. Struct..

